# Overriding a moral default for honesty or dishonesty

**DOI:** 10.1073/pnas.2014489117

**Published:** 2020-08-13

**Authors:** Nobuhito Abe

**Affiliations:** ^a^Kokoro Research Center, Kyoto University, Kyoto 606-8501, Japan

There is a long-standing paradox concerning the cognitive nature of honesty: Is it a matter of “will” or “grace” (1)? The will hypothesis assumes that honesty requires cognitive control to suppress temptation to cheat, while dishonest behavior to serve self-interest is people’s automatic response. In contrast, the grace hypothesis assumes that honesty flows automatically without active resistance to temptation, while dishonest behavior is realized by cognitive control to override honest impulses. The previous findings related to this debate are mixed: Some studies have empirically supported the will hypothesis (2, 3), but others have empirically supported the grace hypothesis (4, 5). In an ambitious study in PNAS, Speer et al. (6) provide reconciliation between these two competing hypotheses, indicating that the prefrontal network could orchestrate both the honesty of individuals who are generally dishonest and the dishonesty of those who are generally honest through cognitive control, which depends on the individual’s moral default.

Speer et al. suggest a reconciliation between the evidence supporting the will and grace hypotheses by focusing on individual differences in honesty, using the newly developed experimental paradigm. In the study of self-serving dishonesty, researchers often engage participants in tasks that enable them to be honest or to cheat to obtain monetary rewards (7). For example, in tasks that reward participants according to their self-reported accuracy of a private prediction of random coin flip, some of the participants report higher-than-chance accuracy, suggesting that they cheat to increase monetary gain. Although this kind of task design is useful to determine whether a participant shows improbably high levels of self-reported accuracy, from which researchers can infer their dishonest behavior, it does not allow the identification of individual lies: Whereas some of the cheating trials involve actual lying, others can be won honestly. This situation is problematic, especially for the analysis of functional neuroimaging data, where researchers want to track on which trials the participants cheated.

To overcome this substantial methodological challenge in identifying individual lies, Speer et al. developed an innovative task, which they call the spot-the-difference task. In this task, participants were presented with pairs of images. Each pair of images included one (25%), two (25%), or three differences (50%), such as objects added to or removed from one of the images or objects with different colors between two images. However, participants were told that all of the pairs included three differences, and they were asked to report whether they could spot all three differences in each trial by providing just yes/no answers without having to point them out. The monetary reward was contingent on participants’ “yes” response so that they could cheat by reporting having found all three differences even when the number of actual differences was one or two. Thus, this task enables inconspicuous measurement of self-serving dishonesty on a trial-by-trial basis.

Speer et al. found large individual differences in the proportion of cheating, from the most honest to the most dishonest, enabling examination of neural correlates of honest and dishonest behaviors that are modulated by the individual’s honesty level. The analysis of functional neuroimaging data yielded several key findings. First, activity in the nucleus accumbens (NAcc), a critical region for reward processing (8), during the decision phase (just before providing yes/no response) significantly predicted the frequency of cheating across participants. A previous neuroimaging study measured reward sensitivity based on activity of the NAcc in response to anticipated rewards and found a positive correlation between NAcc activity and dishonest behavior (9). Speer et al.’s results are an important extension of this work, revealing that the NAcc promotes dishonesty, particularly for participants who cheat frequently.

The results of Speer et al. raise interesting questions about what determines an individual’s moral default with respect to honesty and dishonesty.

Second, participants who are generally honest exhibited increased neural activity in a network consisting of the medial prefrontal cortex (MPFC), posterior cingulate cortex (PCC), and temporoparietal junction (TPJ) when faced with the opportunity to cheat. Prior research indicates that most people cheat a small amount to reap additional rewards while maintaining a positive self-image (10). Speer et al. interpret that particularly honest participants value their moral self-concept and its maintenance through self-referential thinking processes (11). While there remain other possible interpretations for the role of this network, Speer et al. link the MPFC–PCC–TPJ network to the processes for maintenance of positive self-image posited by the current standard theory of dishonesty.

**Fig. 1.**
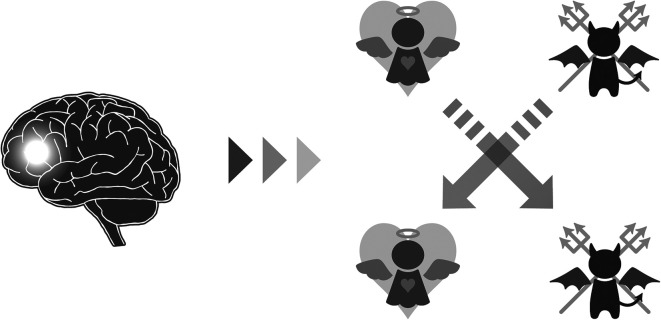
Schematic illustration of the role of cognitive control for overriding moral default proposed by Speer et al. Cognitive control increases cheating in those who have a default inclination to behave honestly, while it promotes honesty in those who have a default inclination to behave dishonestly.

Third, and most importantly, patterns of activity in brain regions responsible for cognitive control differed between those who consistently behaved honestly and those who cheated frequently. Participants who showed a higher rate of cheating exhibited increased brain activation in the anterior cingulate cortex (ACC), a region implicated in conflict detection (12), and the inferior frontal gyrus (IFG), a region necessary for response inhibition (13), when making decisions to be honest. Further analysis on trial-level prediction of cheating revealed that higher activity in the IFG is associated with lower probabilities of cheating in those who cheat frequently, whereas it is associated with higher probability of cheating in those who generally decide to be honest. Thus, the frontal control network is not a simple facilitator or inhibitor of dishonesty. Instead, it plays a flexible role in overriding automatic dispositions to behave honestly or dishonestly (Fig. 1). Speer et al. state that “a generally honest person will need to overcome the default of being honest in order to profit from cheating from time to time, whereas a cheater needs to inhibit the predominant selfish response in order to occasionally be honest and maintain their self-concept.” These findings extend our knowledge about the role of cognitive control, which varies depending on the individual’s moral default, for honest and dishonest moral decision making.

Although Speer et al. provide insights into the role of cognitive control, the exact nature of control-related activity remains unknown. IFG activity is engaged when participants go against their default behavior, but it does not discriminate those who are generally honest deciding to cheat from those who are generally dishonest deciding to be honest. Is overriding “good” default behavior the same as overriding “bad” default behavior at the neural level? It will be important to test whether neural representations of cognitive control associated with nonhabitual honesty differ from those associated with nonhabitual dishonesty using more sensitive analytical methods such as multivariate pattern analysis (14).

The results of Speer et al. raise interesting questions about what determines an individual’s moral default with respect to honesty and dishonesty. Speer et al. speculate that the ACC may encode individual differences in moral default to some extent. This hypothesis is intriguing, given the previous study showing that incarcerated offenders with higher psychopathic traits exhibited reduced ACC activity and reaction times during decisions to cheat, which might be driven by a lack of moral concern about behaving dishonestly (15). Future studies are needed to elucidate how dispositions toward honest or dishonest moral behavior are encoded in the human brain. From a behavioral perspective, measuring implicit attitudes toward dishonesty using tasks such as the implicit association test might provide a clue to better assess individual differences in moral default (16).

It is worth commenting here about the idea that individual differences in honesty are distributed along a continuum. Speer et al. suggest that participants on one side of the spectrum have automatic dispositions to behave honestly, which is associated with more self-referential thinking when faced with the opportunity to cheat. In contrast, participants on the other side of the spectrum have a general tendency to behave dishonestly, and their decisions to cheat are driven more strongly by rewards. This spectrum can be viewed as a continuum from honesty to dishonesty with respect to individual differences in behavioral outputs and is indeed compatible with previously proposed within-honesty and within-dishonesty continuums. That is, a previous study on honesty suggested that relatively weak responses to anticipated rewards predict “graceful” honesty, but individuals who respond more strongly exhibit “willful” honesty, which can be interpreted as the process of overriding the default intuition for dishonesty (9). Likewise, another study on dishonesty reported that lower psychopathic traits predict more controlled or willful dishonesty, which can be interpreted as the process of overriding the impulse of honesty, while higher psychopathic traits are associated with more automatic or “disgraceful” dishonesty (15). Another line of work related to this continuum account is the meta-analysis conducted by Köbis et al. (17), who demonstrated that social harm moderates intuitive honesty and dishonesty. They suggested that only when no concrete other is harmed is people’s intuitive response to make dishonest decisions to serve self-interest. Taken together, these findings indicate that individual differences in honesty and their underlying mechanism constitute a continuum with cognitive control applied to varying degrees, influenced by several moderating factors.

As a final note, I emphasize that the unique approach of the spot-the-difference task adopted by Speer et al. holds promise to deepen our understanding of the cognitive and neural mechanisms underlying honesty and dishonesty. Recent studies have provided new ways to assess human honesty not only in laboratory tasks but also in field experiments, such as multination lost wallet experiments (18). These new lines of studies would jointly illuminate the nature of human honesty from an individual’s neurobiology level to the collective, sociocultural level, possibly leading to the development of effective interventions to reduce self-serving dishonesty.
